# New Process for Anatomical Injections

**Published:** 1843-04

**Authors:** 


*New Process for Anatomical Injections.*—In a letter addressed to the Academy of Sciences, Paris, July 11, 1841, Doyere gives the following account:—I have employed, for nearly two years, a very simple process for obtaining fine injections. This process, which I believe likely to render some service to the anatomy of structure, and probably also to pathological anatomy, essentially consists in causing to enter in the same vessels, *within a certain interval of time*, two finely filtered saline solutions, which, by double decomposition, give an abundant and opaque precipitate. This succession of two injections is that which distinguishes my process from many others tried without success to obtain the injection of the capillary system by the same principle. I inject the second solution, as soon as the first has passed from the arterial system into the venous and lymphatic systems.

I have tried on animals a great number of insoluble salts, with a view to determine those which would give the most satisfactory results. I prefer to all others the chromate of lead. I first inject the chromate of potass, and am convinced that the order of injection is a point not to be neglected. A blue colour may be obtained by the precipitation of Prussian blue; brilliant red by iodide of mercury; white by the carbonate or sulphate of lead. The first has better succeeded with me than the carbonates and sulphates of lime and baryta.

The advantages which this process appears to me to possess over those in use, are above all to shorten the process of making fine injections, and to supersede any other preparation. It may be used with equal advantage cold or hot, in general or partial injection; the materials employed are unalterable, and may be consequently always ready. I will add, that the most minute injections required only a pressure which was evidently less than that of the heart's action. Poiseuille, to whom I made the process known several months since, in order that he might make use of it in his particular researches, has constructed an instrument by the assistance of which he can inject either liquid with that degree of pressure he considers proper.

By the assistance of this process, I have more than once succeeded in injecting by the femoral artery in a single operation, and in a few minutes, the capillaries of the muscular system in an entire animal, the adipose and cellular systems of the white and gray matter of the brain, of the conjunctiva, of all the mucous membranes, intestinal villosities, &c. The capillaries thus injected by the chromate of lead are more filled, especially after drying, than by the injections of size, but less than by those of varnish (*vernis*); there also remains some doubt in my mind relative to the actual diameter of the latter canals. Those which run parallel to each primitive muscular fasciculus, to the number of four or six, appeared to me to possess, in the dog, $\frac{1}{400}$th or $\frac{1}{300}$ of a millimetre; but it is possible that their dimensions had been reduced by the action of one or the other of the two solutions employed, or that they had not been sufficiently filled. I am now engaged in determining the relation which exists between the size of injected vessels, and their size during life.—*Microscopic Journal* from *Comptes Rendus*, July 1841.

